# Strategien für die Schaftrevision: Operationsplanung, Implantatentfernung und Reimplantation

**DOI:** 10.1007/s00132-022-04277-y

**Published:** 2022-07-08

**Authors:** Sebastian Hardt, Lukas Schönnagel, Christian Hipfl

**Affiliations:** Centrum für Muskuloskeletale Chirurgie, Charité – Universitätsmedizin Berlin, Charitéplatz 1, 10117 Berlin, Deutschland

**Keywords:** Schaftwechsel, Osteotomie, Prothesenlockerung, Hüftrevision, Totaler Hüftgelenkersatz, Stem revision, Osteotomy, Prosthesis loosening, Hip revision surgery, Total hip replacement

## Abstract

**Hintergrund und Planung:**

Die Schaftrevision stellt in ihrer Operationsvorbereitung und technischen Durchführung eine große Herausforderung dar. Die Ergebnisse sind maßgeblich von der Defektsituation, der Qualität der Implantatentfernung sowie der Wahl des Revisionsimplantates abhängig. Patientenspezifische Faktoren wie das Alter, die Komorbiditäten, die Knochenqualität oder auch die Lokalisation des Zementes haben entscheidenden Einfluss auf die operative Strategie. Eine entsprechende präoperative Vorbereitung inklusive des Vorhandenseins von notwendigen Spezialinstrumenten, die essenziell für die schonende Implantatentfernung sind, ist unabdingbar, um das bestmögliche Ergebnis zu erzielen.

**Therapie:**

Die knochenschonende Explantation stellt gerade bei festsitzenden Schäften und Zementresten, die ggf. weit über den Isthmus reichen können, eine besondere Herausforderung dar. In solchen Situationen sollte ein transfemoraler Zugang erwogen werden. Die zementfreie Reimplantation unter Verwendung von modularen oder nichtmodularen Titanschäften ist für die meisten Revisionen die derzeit bevorzugte Therapie der Wahl. Bei älteren Patienten mit niedrigem Leistungsanspruch oder schlechter Knochenqualität bleibt die zementierte Versorgung eine gute alternative Therapieoption.

## Abstract (en)

**Background and planning:**

Femoral revision represents a challenging procedure in its surgical planning and technical execution. The results are mainly dependent on the bone loss present, the quality of the implant removal and the choice of revision system. Patient-specific factors such as age, comorbidities, bone quality or even the localization of the cement have a decisive influence on the surgical strategy. Appropriate preoperative preparation, including the presence of the necessary special instruments, which are essential for gentle implant removal, is indispensable to achieve the best possible result.

**Therapy:**

A bone-sparing implant removal can be particularly challenging, especially in the case of well-fixed stems and cement residues, which may extend far beyond the isthmus. In such situations, a transfemoral approach should be considered. Cementless reimplantation using modular or non-modular titanium stems is currently the preferred treatment of choice for most revisions. In elderly, low-demand patients or with poor bone quality, cemented revision remains a good alternative treatment option.

Die Schaftrevision ist ein anspruchsvolles und aufwändiges Verfahren, dass einer umfangreichen und sorgfältigen Planung bedarf. Das Ergebnis wird durch implantatspezifische und patientenspezifische Faktoren beeinflusst. Von besonderer Bedeutung ist die knochenschonende Explantation. Im Folgenden sollen Einzelheiten zur Diagnostik und Operationsplanung, zu der Vielzahl an Explantationsinstrumenten, den Zugängen für die Implantatentfernung und zum Wechsel dargestellt werden.

## Epidemiologie und Ätiologie

Die häufigsten Indikationen für eine Schaftrevision sind die aseptische Lockerung, die periprothetische Infektion (PPI), die periprothetische Fraktur (PPF), die Luxation, die Malposition sowie das Implantatversagen (Tab. 1). In Deutschland wurden im Jahr 2021 ca. 17.200 Revisionen einer Hüftendoprothese durchgeführt. Davon wurden ca. 28 % (4251 Fälle) komplett gewechselt, bei ca. 22 % erfolgte ein isolierter Schaftwechsel [20].

**Table 1:** 

Indikation zur Schaftrevision
Aseptische Lockerung
Periprothetische Infektion
Periprothetische Fraktur
Luxation
Implantatfehlpositionierung
Metallose
Implantatbruch

Eine aseptische Schaftlockerungen ist am häufigsten durch abriebinduzierte Osteolysen, seltener durch eine Alterung des Knochenzementmantels, eine PPF oder eine „adverse reactions to metal debris“ (ARMD) bedingt [4]. Die aseptische Schaftlockerung ist mit fast 12 % die häufigste Indikation für einen Wechsel der Femurkomponente [20]. In Registerdaten aus Schweden, Großbritannien, Australien und Nordamerika ist die aseptische Lockerung ebenfalls die häufigste Revisionsursache, wobei nicht zwischen Schaft- und Pfannenlockerung unterschieden wird [6, 7, 9, 31].

Durch die in den letzten Jahren deutlich verbesserte Diagnostik bzgl. einer PPI nimmt auch der Anteil septisch bedingter Indikationen zur Schaftrevision zu. Hinzu kommt der steigende Anteil an PPF, welcher durch den demographischen Wandel und der damit verbundenen immer älter werdenden Bevölkerung mit einem immer höher werdenden Mobilitätsanspruch einhergeht.

Der Wechsel eines festsitzenden Schaftimplantates beispielsweise bei Malposition, ARMD oder Instabilität stellt eine besondere Herausforderung bzgl. der Planung und Durchführung des Eingriffs dar.

## Präoperative Diagnostik

Für eine mögliche Revisionsindikation ist eine entsprechende Anamnese mit klinischer Untersuchung und konventioneller Bildgebung essenziell. Insbesondere bei einer Frühlockerung (≤ 5 Jahre) sollte auch bei unauffälligen Laborwerten die Indikation für eine präoperative Punktion (Bestimmung der Leukozytenzellzahl sowie mikrobiologische Untersuchungen) zum Ausschluss einer PPI gestellt werden [45].

Bei einer Frühlockerung ist eine präoperative Punktion obligat

Ein Röntgenbild in 2 Ebenen mit Abbildung der kompletten Prothese bzw. des eventuell weit nach distal reichenden Zementköchers ist für die Planung des operativen Vorgehens unbedingt erforderlich [40]. Zeichen für eine Schaftlockerung sind ein Lockerungssaum größer als 2 mm, eine progrediente Migration des Schaftes, eine Hypertrophie der Kortikalis oder die Fraktur des Zementmantels [29, 46]. Die Lokalisation von Lysesäumen kann nach Gruen in 14 Zonen eingeteilt werden [25]. Bei unklaren Befunden kann die radiologische Diagnostik durch eine Computertomographie ergänzt werden [46]. Diese kann bei der Beurteilung von Osteolysen und Knochendefekten hilfreich sein und auch zur Bestimmung einer Malrotation der Schaftkomponente genutzt werden [53, 67].

Bei Verdacht auf eine ARMD ist eine MRT mit Metall-Artefakt-Suppression (MARS-MRT) zur Abklärung eines Pseudotumors indiziert [15, 44]. In Abhängigkeit von der verwendeten Gleitpaarung, der Modularität des Schaftes oder bei Verdacht auf eine Konuskorrosion sollte die Serumionenkonzentrationen von Kobalt und Chrom bestimmt werden.

**Figure 1:**
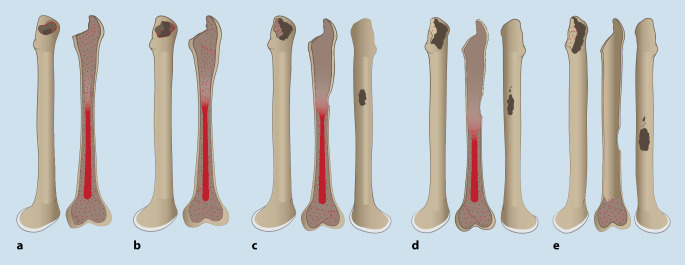

Die femorale Defektsituation sollte präoperativ nach Della Valle und Paprosky (Abb. 1) klassifiziert werden [62]. Davon abhängig kann eine entsprechende Implantatwahl für die Revisionsoperation getroffen werden.

## Operationsplanung

### Patientenspezifische Faktoren

Alter, Vorerkrankungen, Voroperationen, Knochenqualität und die Lebenserwartung sind in das therapeutische Vorgehen mit einzubeziehen. Beispielweise sind ein hohes Patientenalter, eine bekannte Sturzneigung und schlechte Knochenqualität ausschlaggebend, um eine zementierte Schaftrevision zu präferieren, da hiermit das peri- und postoperative Frakturrisiko reduziert werden kann und eine schnelle Vollbelastung möglich ist [1]. Als wichtige modifizierbare Faktoren sind ein Diabetes (Hämoglobin [Hb] A1c > 8), Adipositas, Rauchen, Anämie und Hypoalbuminämie (Serumalbumin < 3,5 g/dl) zu nennen [8, 10, 24, 37, 54]. Diese gilt es präoperativ zu erfassen und wenn möglich im Rahmen einer Prähabilitation zu optimieren, um das Komplikationsrisiko zu reduzieren.

Vor der Operation sollten zusätzlich das aktuelle Aktivitätsniveau und bestehende Einschränkungen des Patienten mittels standardisierter Fragebögen (z. B. Harris-Hip-Score) erfasst werden, um den Therapieerfolg einschätzen und realistische Therapieziele setzen zu können.

Die Zahl der Voroperationen und der verwendete Zugangsweg spielen ebenfalls eine wesentliche Rolle für das zu erwartende Outcome nach Schaftrevision [11, 36]. Eine vorbestehende Glutealinsuffizienz ist mit großer Wahrscheinlichkeit mit einer niedrigeren Patientenzufriedenheit vergesellschaftet und hat einen Einfluss auf das postoperative Luxationsrisiko [19].

### Prothesenspezifische Faktoren

**Figure 2:**
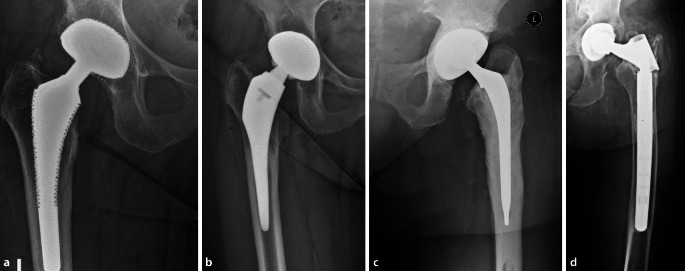

Wenn möglich, sollte der zu explantierende Prothesentyp bekannt sein, um ggf. implantatspezifische Instrumente zum Ausbau bereit zu haben. Schäfte mit einer makrostrukturierten Oberfläche oder modulare Steckkonen, für die kein Explantationsinstrumentarium verfügbar ist, zählen zu den schwer zu explantierenden Implantaten (Abb. 2).

Vor der Operation sollte eine digitale Planung erfolgen, um eine möglichst genaue Rekonstruktion der Biomechanik zu erzielen und die Prothesengröße abschätzen zu können.

### Spezielle Explantationsinstrumente

Eine möglichst knochen- als auch weichteilschonende Entfernung des alten Implantats sollte das Ziel jeder Revision sein. Hierzu können verschiedene Instrumente zu Hilfe genommen werden:

#### Ausschlaginstrumente.

Häufig können Universalausschläger, welche an dem Konus des Schaftes greifen, verwendet werden. Einige Prothesen verfügen über Gewinde, in die ein entsprechender Ausschläger geschraubt werden kann.

#### Meißel.

Bei jeder Revisionsoperation sollte ein großes Sortiment an Meißeln in verschiedenen Stärken, Längen und Biegungen vorhanden sein. Flexible Whelan-Meißel haben sich etabliert, um das Interface zwischen Prothese/Zement und Knochen aufzubrechen. Weiterhin können pneumatische Meißel zur Entfernung von Zement oder zum Ummeißeln festsitzender Schäfte genutzt werden.

#### Kirschner-Drähte.

Alternativ zu entsprechenden Meißeln können auch einfache K‑Drähte (2,0 mm) zum Aufbrechen des Interfaces zwischen Prothese/Zement und Knochen genutzt werden. Diese können gerade im Bereich des Kalkar hilfreich sein und weisen eine deutlich geringere „Sprengwirkung“ als herkömmliche Meißel auf.

#### Fräsen.

Hochfrequenz-Fräsen (z. B. Midas Rex® [Medtronic, Meerbusch, Deutschland]) sind bei knöchern sehr gut integrierten Schäften bzw. sehr gut verzahntem Zement hilfreich und ermöglichen eine möglichst knochenschonende Explantation.

#### Bohrer.

Markraumbohrer mit Größen von 6–15 mm mit 1 mm Abstufungen sowie Bohrer mit nichtschneidenden Spitzen und Zentrierhülsen zur Entfernung von Zementresten sollten vorhanden sein. Kanülierte Bohrer können in Seldinger-Technik benutzt werden, um leichter in den distalen Markraum zu gelangen.

Um eine endofemorale Entfernung von Knochenzement zu vereinfachen, existieren beispielsweise langstielige Greifzangen oder entsprechend lange Haken- und Korkenzieher sowie Küretten, um Zementreste bergen und den Markraum debridieren zu können. In selten Fällen können chirurgische Ultraschallgeräte zur Entfernung von Knochenzement hilfreich sein. Hierbei werden sowohl senkrechte als auch Torsionsbewegungen der Sonde zur Verflüssigung des Zements genutzt. Neuere Geräte geben bei kortikalem Kontakt ein akustisches Signal, wodurch das Risiko für Perforationen verringert werden soll. Bei der Extraktion eines gebrochenen Schaftes können zusätzliche Spezialwerkzeuge sinnvoll sein. Entsprechende Gewindeschneider, Hohlfräsen in verschiedenen Längen und Durchmessern sowie Trepanfräsen können in diesen Fällen nützlich sein.

### Zugänge

Der operative Zugang hat einen wesentlichen Einfluss auf die postoperative Funktion und Zufriedenheit des Patienten. Bisher existieren nur wenige wissenschaftliche Erkenntnisse zur Zugangswahl bei Revisionseingriffen. Der Wechsel des primären Zugangs scheint hinsichtlich der Komplikationsrate keinen Unterschied zu machen [26]. Hinsichtlich der postoperativen Funktion und Patientenzufriedenheit gibt es keine Daten. Grundsätzlich sollte daher für die Schaftrevision der Zugang gewählt werden, mit welchem der Operateur am besten vertraut ist. Der gewählte Zugang muss aber eine ausreichende Übersicht ermöglichen und vor allem gut erweiterbar sein.

Der posteriore Zugang wird weltweit sowohl in der Primärendoprothetik als auch bei Revisionen am häufigsten verwendet [32]. Er bietet eine exzellente Exposition sowohl femoral als auch azetabulär und ist nach distal einfach zu erweitern. Bei zusätzlicher Pfannenrevision mit ausgedehnter Defektsituation und/oder Beckendiskontinuität bietet der posteriore Zugang außerdem eine adäquate Übersicht des dorsalen Pfeilers. Der größte Nachteil ist die dauerhafte Schädigung der Außenrotatoren, was das Luxationsrisiko erhöht [13, 50]. Die Rekonstruktion der posterioren Kapsel und der Außenrotatoren während der Primärimplantation verringert das Luxationsrisiko, ist aber bei Revisionseingriffen nicht immer durchführbar [58].

Der anterolaterale Zugang bietet auch im Revisionsfall den Vorteil, dass die Glutealmuskulatur weitestgehend geschont werden kann. Der Patient kann sowohl auf dem Rücken als auch auf der Seite liegen. Die distale Erweiterung durch einen Subvastuszugang erlaubt eine sehr gute Darstellung des Femurs und ist chirurgisch einfach durchzuführen.

Auch der laterale (transgluteale) Zugang erlaubt eine übersichtliche Darstellung des Femurs. Er entspricht im Wesentlichen der Technik der Primärimplantation. Nachteil ist die nochmalige Schädigung des M. gluteus medius, weshalb dieser Zugang für Revisionseingriffe nur selten indiziert ist [64].

Nur von wenigen Autoren wird der anteriore Zugang zur Schaftrevision genutzt [48]. Aus unserer Sicht sollte dieser aufgrund der eingeschränkten Erweiterbarkeit für komplexe Schaftrevisionen nicht genutzt werden. Sollte ein transfemoraler Zugang notwendig sein, muss entweder eine zusätzliche seitliche Inzision durchgeführt oder der Schnitt in einem Bogen zur lateralen Seite des Oberschenkels verlängert werden [38].

#### Endofemoraler Zugang

Die endofemorale Explantation stellt bei einer Großzahl der Schaftrevisionen die erste Wahl dar. Gelockerte Schäfte können nach Freilegen der Prothesenschulter durch einen entsprechenden Ausschläger problemlos entfernt werden. Bei gut osseointegrierten Schäften oder sehr gut verzahntem Zement müssen spezielle Techniken und Instrumente genutzt werden, welche im Abschnitt Implantatentfernung erläutert werden.

#### Transfemoraler Zugang

Durch das Anlegen einer erweiterten Trochanterosteotomie oder Wagner-Osteotomie kann die Explantation eines festsitzenden Schaftes oder Knochenzementes vereinfacht beziehungsweise überhaupt erst möglich werden. Die Osteotomielänge hängt von der Länge des zu entfernenden Schaftes oder Zementmantels ab. Grundsätzlich gilt, dass der transfemorale Zugang so kurz wie notwendig gewählt werden sollte, um die Notwendigkeit von langen Revisionsschäften zu vermeiden.

Insbesondere bei einem makrostrukturierten, einem den Markraum komplett ausfüllenden oder einem langstreckig, osseointegrierten Schaft, einem weit nach distal reichenden, gut verzahnten Zementköcher oder sehr schlechter Knochenqualität mit kortikaler Ausdünnung < 2 mm empfiehlt sich die primäre Anwendung eines transfemoralen Zugangs (Tab. 2), um eine Perforation oder Fraktur zu vermeiden. Bei gebrochenen oder modularen Schäften ohne passendes Explantationsinstrumentarium oder ausgedehnten Osteolysen oder periprothetischen Frakturen ist ebenfalls ein transfemoraler Zugang sinnvoll, um weiteren Knochenverlust oder Frakturen zu verhindern.

**Table 2:** 

Indikation zum transfemoralen Zugang
*Zementfreier Schaft*
Makrostrukturierter Schaft
Fest integrierter Langschaft
Gebrochener Schaft
Modularer Schaft mit fehlendem Explantationsinstrumentarium
Langstreckig diaphysärer Knochenverlust
Deformität oder fehlverheilte Fraktur proximales Femur
*Zementierter Schaft*
Festsitzender Zement
Langstreckig diaphysärer Knochenverlust
*Schwierige oder riskante Prothesenluxation*
Ausgeprägte periartikuläre Ossifikationen oder Ankylose
Tiefsitzender Schaft
Ausgeprägte Fragilität Trochanter major

Im Falle mehrerer frustraner Versuche kann eine longitudinale Split-Osteotomie hilfreich sein

Im Falle mehrerer frustraner Versuche der endofemoralen Entfernung kann eine longitudinale *Split-Osteotomie* hilfreich sein. Bei einem posterioren Zugang wird dabei eine Osteotomie in einer Linie mit dem hinteren Rand des Vastus lateralis knapp lateral der Linea aspera angelegt. Die Osteotomie wird üblicherweise mit einer oszillierenden Säge durchgeführt und reicht vom metadiaphysären Übergang je nach Länge des Schaftes bis etwa 5 cm nach distal. Bei einem lateralen Zugang wird die Osteotomie analog an der Ventralseite des Femurs angelegt. Mit einem entsprechenden Osteotom wird eine lokale Ablösung des Knochens von der porösen Schaftoberfläche erreicht. Falls trotz einer Split-Osteotomie keine Schaftextraktion erzielt werden kann, können die jeweiligen Osteotomien einfach zu einem entsprechenden transfemoralen Zugang erweitert werden.

**Figure 3:**
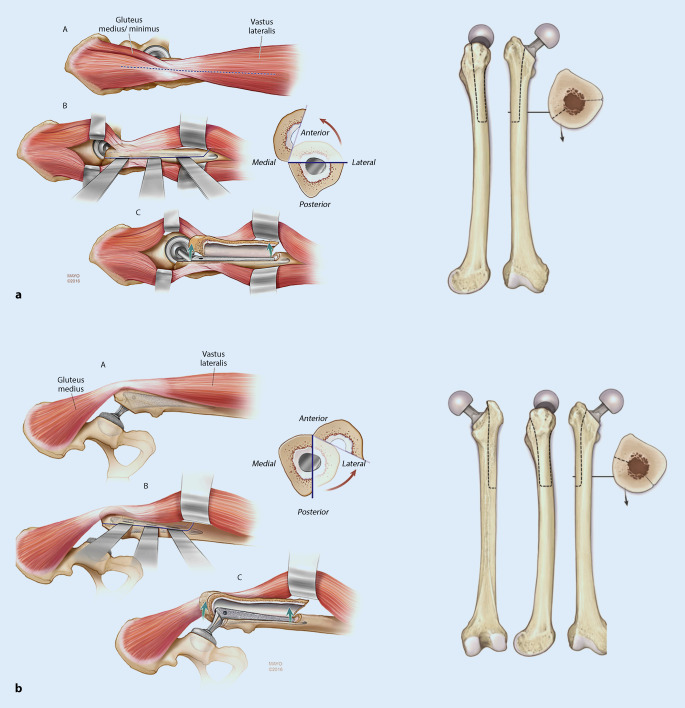

Bei Anwendung eines lateralen Zugangs wird das Femur über eine anterolaterale Osteotomie eröffnet, der sogenannten *Wagner-Osteotomie* (Abb. 3a).

Entscheidende Weichteilstrukturen müssen erhalten bleiben, insbesondere der Verbund zwischen Vastus lateralis und Gluteus medius, um die Durchblutung des entstehenden Knochendeckels nicht zu gefährden. Zunächst muss der Vastus lateralis nach distal bis auf Höhe des geplanten transversalen Schnitts gespalten werden. Die Höhe und Länge des transversalen Schnitts werden mit Bohrlöchern festgelegt. Der transversale Schnitt und somit die Breite des Knochendeckels sollte nicht mehr als ein Drittel des Umfangs der Oberschenkeldiaphyse umfassen. Anschließend wird das Femur lateralseitig mit einer Säge am vordefinierten Weg von proximal nach distal osteotomiert. Der transversale Schnitt wird entweder mit einer Hochgeschwindigkeitsfräse oder einem schmalen Sägeblatt durchgeführt. Der ventrale Schnitt erfolgt mit einer Säge.

Bei einem posterioren Zugang wird das Femur durch eine dorsolaterale Osteotomie eröffnet (Abb. 3b). Der transfemorale Zugang wird auch hier in drei Phasen durchgeführt: direkte Osteotomie dorsalseitig an der Linea aspera, transversaler Schnitt und ventrale Eröffnung, meist mit dem Meißel. Zunächst wird der hintere Rand der Linea aspera freigelegt, nach distal über einen Subvastuszugang. Die Länge des Knochenfensters ist ebenfalls von dem zu explantierenden Schaft bzw. dem Zementköcher abhängig, und liegt typischerweise bei 12–15 cm [59]. Der ventrale Schnitt wird wieder zunächst distal oberhalb der Ebene des transversalen Schnittes mit dem Meißel oder der Säge begonnen und mit dem Meißel nach proximal zwischen Prothesenhals und Trochanter minor komplettiert. Anschließend wird durch das Einführen von Osteotomen am dorsalen Schnitt der Knochendeckel abgehoben und nach ventral verlagert.

## Implantatentfernung

### Explantation zementfreier Schäfte

Die Schaftexplantation ist anspruchsvoll und die spätere Verankerung hängt maßgeblich von der Qualität des Ausbaus ab.

**Figure 4:**
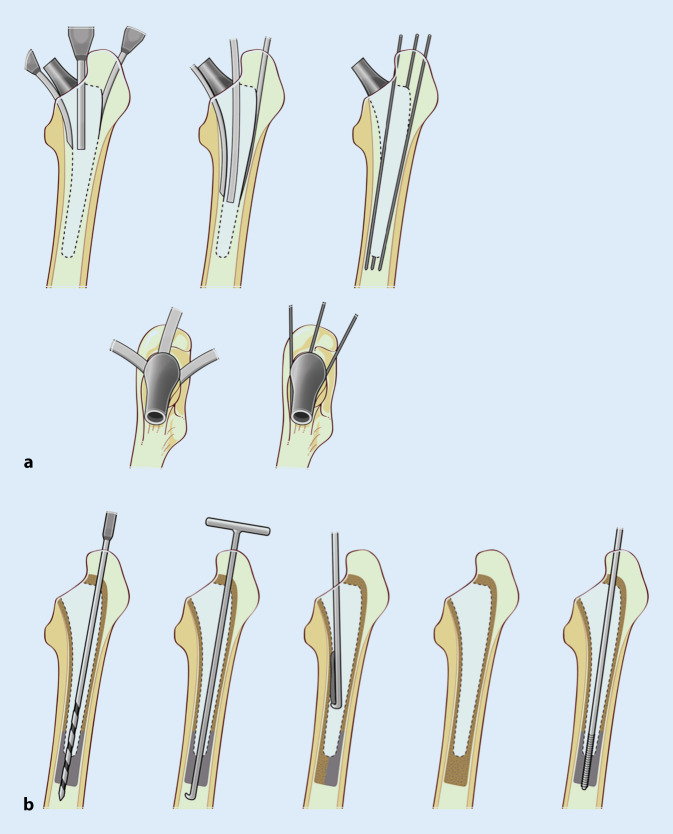

Kurz- und Standardschäfte können in vielen Fällen endofemoral entfernt werden. Zunächst sollte das über der Prothesenschulter befindliche Granulations- und Knochengewebe ausreichend entfernt und der Kalkar freigelegt werden, um das Frakturrisiko im trochantären Bereich zu reduzieren. Anschließend können die oben beschriebenen Instrumente genutzt werden, um ein knochenschonendes Aufbrechen des Interfaces zwischen Knochen und Implantat zu ermöglichen. Je weiter distal man zur Schaftspitze vordringt, desto längere und schmalere Meißel werden verwendet. (Abb. 4a). Anschließend wird versucht, den Schaft mit einem passenden Extraktionssystem zu entfernen. Andernfalls kann ein universelles Extraktionssystem, welches sich meistens am Prothesenhals verklemmt, verwendet werden. Die Möglichkeit zum Ansetzen des Ausschlaginstrumentariums direkt am Schaft erhöht den Erfolg einer endofemoralen Explantation durch die direkte Krafteinleitung maßgeblich.

Zur Entfernung gebrochener Schäfte können, wie oben beschrieben, Kronenbohrer oder Trepane genutzt werden. Wichtig dabei ist es, durch kontinuierliche Spülung thermisch induzierte Nekrosen des Knochens zu minimieren [30].

### Explantation zementierter Schäfte

Zementierte Schäfte lassen sich in der Regel problemlos endofemoral entfernen. Die größte Herausforderung besteht hier in der Entfernung des Zements und des Stoppers. Bei fest verzahntem Zementköcher, welcher zusätzlich über den Isthmus reicht, kann sich die Entfernung häufig frustran gestalten. In aseptischen Situationen sollte an eine Zement-in-Zement-Revision gedacht werden (s. unten). Zu den Komplikationen gehören Perforationen, Frakturen und unvollständige Zemententfernung, die die Reimplantation eines Revisionsschaftes maßgeblich beeinflussen [60].

Schlanke, gerade Meißel sollten mit Vorsicht verwendet werden, um mögliche Perforationen zu vermeiden. Haken und Küretten dienen dazu, den Femurkanal oder Zementreste zu ertasten und gelockerte Fragmente zu entfernen. Durch sukzessives Aufbohren können distal gelegene, fest verzahnte Zementreste entfernt werden. Der Zementstopper wird zentral überbohrt und mittels Haken- oder Korkenzieherküretten entfernt (Abb. 4b). Insbesondere bei exzentrischer Schaftlage kann das Aufbohren zur Ausdünnung der Kortikalis und ggf. zu einer Perforation oder Fraktur führen. Eine kontinuierliche fluoroskopische Kontrolle in 2 Ebenen ist in diesen Fällen zu empfehlen.

Bei einem langstreckig fest verzahnten oder einem weit über den Isthmus reichenden Zementköcher ist die Indikation für einen transfemoralen Zugang großzügig zu stellen, um eine kontrollierte Zemententfernung zu gewährleisten und das Frakturrisiko zu minimieren.

Bei einem langstreckig fest verzahnten Zementköcher ist die Indikation für einen transfemoralen Zugang großzügig zu stellen

Die Anlage eines *Knochenfensters* bietet sich insbesondere an, um Knochenzement, Zementstopper oder anderes Fremdmaterial in der Femurdiaphyse zu entfernen. Es sollte darauf geachtet werden, dass dieses an der mechanisch weniger beanspruchten Ventralseite angelegt wird. Die Ecken des Knochenfensters werden wieder mit Bohrlöchern festgelegt, wodurch Fissuren vermieden werden können. Anschließend wird der Deckel mit einer kleinen Säge oder Meißeln keilförmig aus der Kortikalis gelöst. Das Knochenfenster sowie die durch transfemorale Zugänge entstandenen Knochendeckel werden mit Cerclagen refixiert. Die Split-Osteotomie wird ebenfalls mit einer Cerclage gesichert. Der Revisionsschaft sollte die Osteotomie je nach Technik und Implantatwahl um mindestens 3–5 cm überbrücken, um einen Ermüdungsbruch zu vermeiden und eine ausreichende Stabilität zu generieren.

## Reimplantation

### Zementfreie Reimplantation

Prinzipiell ist eine zementfreie oder zementierte Reimplantation möglich, wobei aus unserer Sicht der Einsatz einer zementierten Technik weitaus komplizierter ist und häufig eine aufwendige Spongiosaplastik erfordert. Daher ist es erklärlich, dass weltweit zunehmend zementfreie Revisionsschäfte zur Anwendung kommen.

**Figure 5:**
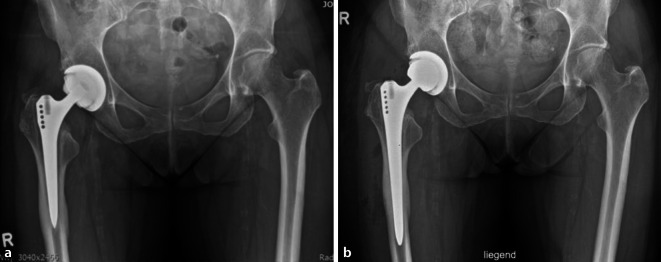

Historisch waren nicht modulare, sog. „Fully-porous-coated“-Schäfte mit zylindrischem Querschnitt das Implantat der Wahl für die meisten Femurrevisionen [18]. Geradschäfte mit rechteckigem Querschnitt zeigen bei Hüften mit Typ-I- bis -IIIa-Defekten ebenfalls gute, langfristige Standzeiten (Abb. 5a,b; [35]). Hierbei ist die Morphologie des proximalen Femurs nicht entscheidend, da die Fixierung im Bereich der Diaphyse bzw. des Isthmus erfolgt.

Vor allem bei Typ-I- und Typ-II-Defekten zeigen diese Implantate eine sehr gute Primärstabilität. Bei IIIa-Defekten ist die zu erzielende Stabilität maßgeblich von dem zu erreichenden flächigen Kontakt mit dem Knochen (mind. 3 cm) abhängig. Häufig gibt die veränderte Anatomie des proximalen Femurs die Rotation vor. Hierdurch ist auf ein mögliches Implantatimpingement und damit eine potenzielle Instabilität zu achten. Die Gefahr einer periprothetischen Fraktur ist als weiterer Nachteil von zylindrischen oder rechteckigen Standard- beziehungsweise Revisionsschäften bei IIIa-Defekten zu nennen [18]. Um diese Probleme zu adressieren, haben sich über die letzten zwei Jahrzehnte modulare Titanschäfte mit sternförmigem Querschnitt bei größeren Defekten etabliert. Hierdurch kann auch bei größeren Defekten (Typ IIIb und IV) mit kurzer diaphysärer Verankerungsstrecke ein guter Implantat-Knochen-Kontakt erreicht werden. Die Modularität ermöglicht im nächsten Schritt die Rekonstruktion der Beinlänge, der femoralen Anteversion und des femoralen Offsets.

Die Hauptnachteile von modularen Revisionsschäften sind das Risiko eines Versagens der modularen Verbindung, die Kosten des Implantats sowie technisch schwierigere Anwendung [47]. Länge, Offset, Anteversion und Durchmesser des proximalen Teils sind entscheidende Einflussgrößen auf die entstehenden Kräfte an der modularen Verbindung und somit für das Risiko eines Materialversagen. Auch ein hoher Body-Mass-Index, ein hohes Aktivitätsniveau des Patienten sowie eine reduzierte proximale Knochensubstanz erhöhen das Risiko für das Implantatversagen im Bereich der Modularität [33].

Die „ideale“ proximale Komponente eines modularen Revisionsschafts sollte eine Länge von 70–90 mm haben

**Figure 6:**
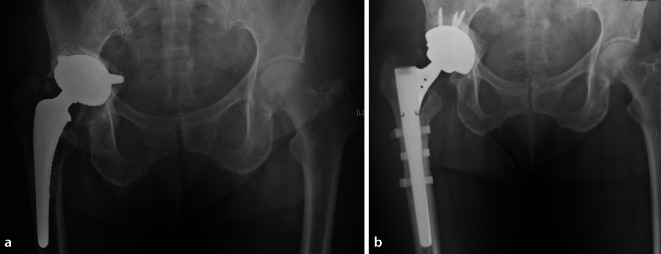

**Figure 7:**
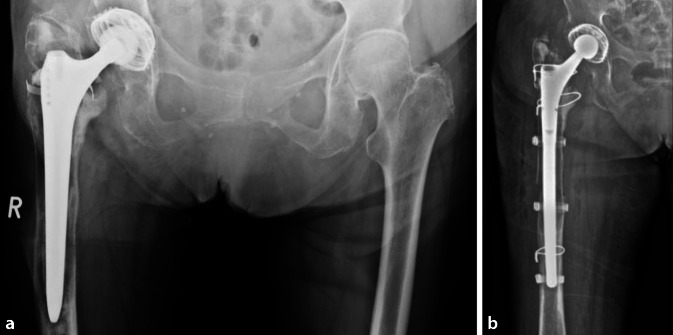

Prinzipiell kann ein gerader oder ein kurvierter Schaft verwendet werden. Aus unserer Erfahrung ist bei Schaftlängen bis 20 cm ein gerader Schaft zu bevorzugen (Abb. 6a,b). In Abhängigkeit von der Kortikalisdicke beziehungsweise der Knochenqualität sollte auf einer diaphysären Strecke von mindestens 3 cm eine stabile Press-fit-Verankerung des Schaftkonus erreicht werden. Biomechanische Untersuchungen zeigen, dass die „ideale“ proximale Komponente eine Länge von 70–90 mm haben sollte, um die Bruchgefahr im Bereich der Modularität zu reduzieren [28]. Natürlich spielt aber diesbezüglich die genaue Defektmorphologie eine wesentliche Rolle. Bei längeren Schäften mit nur sehr kurzem Isthmus (Typ-IIIb- und -IV-Defekte), nach transfemoralem Zugang mit langem Fenster oder bei periprothetischen Frakturen, sind kurvierte Schäfte indiziert (Abb. 7a,b). Prinzipiell spricht man bei kurvierten Schäften von einer 3‑Flächen-Verankerung. Bei einem transfemoralen Zugang wird durch ein Press-fit im Isthmus die Primärstabilität erzeugt und der Knochendeckel nur an den Schaft durch Cerclagen wiederangelegt. Um eine periprothetische Femurfraktur zu vermeiden, empfehlen wir die Anlage einer protektiven Sicherungscerclage um den intakten Isthmus. Durch die Eigenelastizität des Knochens kann die Prothese meist nach kurzem Abwarten noch weiter eingeschlagen werden, wodurch das Risiko eines Undersizing und späteren Schaftmigration reduziert wird.

**Figure 8:**
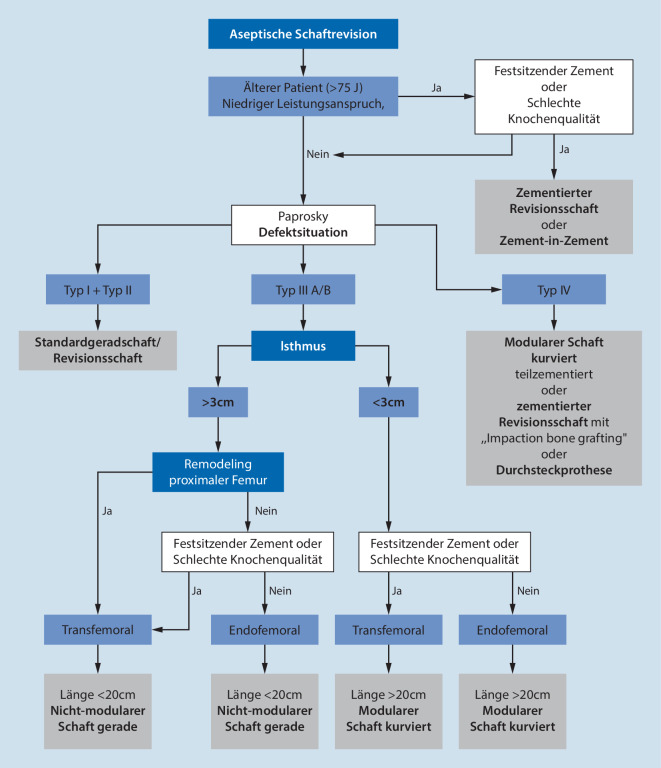

Ist weder eine Press-fit-Verankerung noch eine 3‑Flächen-Verankerung im defizitären Femur zu erreichen, kann eine zementierte Versorgung mittels „impaction bone graftig“ oder eine zusätzliche Verwendung struktureller Allografts (sog. „strut grafts“) erwogen werden. „Impaction bone grafting“ ist allerdings sehr zeitaufwendig und bei großem segmentalem Knochenverlust technisch unmöglich. Bei diesen Typ-IV-Defekten kann ein modularer Revisionsschaft distal zusätzlich mit Verriegelungsschrauben oder mit Zement („hybrid“ – Off-Label ist hierbei zu prüfen) fixiert werden. Sollte die Diaphyse nicht mehr für eine sichere Verankerung ausreichen, ist eine Rekonstruktion nur mittels einer Megaprothese (totaler Femurersatz) beziehungsweise einer Durchsteckprothese möglich. Ein Algorithmus zum therapeutischen Vorgehen ist in Abb. 8 zusammengefasst.

### Zementierte Reimplantation

Bei älteren Patienten mit geringem Leistungsanspruch, einer defizitären Knochenstruktur, bei der ein ausreichendes Press-fit nicht erzielt werden kann und ein hohes Frakturrisiko besteht, ist die Indikation für eine zementierte Reimplantation gegeben. Das Vorhandensein von Spongiosa zur Verzahnung des Zements ist eine unabdingbare Voraussetzung für eine gute Verankerung und Langlebigkeit. Femorale Defekte (z. B. nach Perforation bei ausgewanderten Schäften oder im Rahmen der Schaftentfernung) sind risikobehaftet und reduzieren die Verankerungsqualität. Alle wichtigen Punkte der modernen Zementiertechnik sind zu beachten. Potenzielle Vorteile der zementierten Schaftrevision sind die relative Freiheit der Implantatpositionierung und die Möglichkeit der lokalen Antibiotikabeimischung.

Eine spezielle Operationstechnik stellt das Zementieren in einen vorhandenen Zementköcher dar. Diese sog. Zement-in-Zement-Technik setzt voraus, dass ein stabiler und korrekter, d. h. zentrisch gelegener prothesenumfassender Zementmantel vorhanden ist. Es stellt eine gute Alternative bei älteren Patienten mit fest verzahntem Zementköcher dar, bei welchen der Wechsel auf ein zementfreies Implantat zu zeitaufwendig und das perioperative Risiko unnötig erhöhen würde. Für das Wiedereinzementieren sollte niedrigvisköser Zement verwendet werden.

## Komplikationen und Ergebnisse

Häufige Komplikationen nach Schaftrevisionen sind intra- und postoperative Frakturen, periprothetische Infektionen, aseptische Lockerungen, Schaftmigrationen und Luxationen. Weiterhin führen postoperative Schmerzen, Nerven- und Muskelschädigungen zu einer wesentlichen Einschränkung der Lebensqualität des Patienten. Die Rate an postoperativen Komplikationen ist neben dem Gesamtzustand des Patienten wesentlich von der Revisionsindikation, dem Knochendefekt und dem verwendeten Schaftsystem abhängig.

Standardschäfte zeigen bei Paprosky-Grad-I- und -II-Defekten gute kurz- und mittelfristige Ergebnisse, mit einer Überlebensrate von 95,6 % nach 4,7 Jahren. Die Hauptkomplikationen waren perioperative Frakturen (5,6 %), Dislokationen (5,4 %), Infektionen (2,6 %) und aseptische Lockerungen (1,4 %) [12]. Romagnoli et al. konnten bei einer Serie von 91 Schaftrevisionen auch gute mittelfristige Ergebnisse mit einer Überlebensrate von 95,6 % nach 10 Jahren zeigen [52]. Bei modularen und nichtmodularen Revisionsschäften zeigen sich revisionsfreie Überlebensraten von über 90 % nach 10 Jahren, wobei modulare Revisionsschäfte häufiger bei größeren Knochendefekten zur Anwendung kommen, was den Vergleich erschwert. Die Literatur zeigt bei nichtmodularen Revisionsschäften im Vergleich zu modularen Schäften ein erhöhtes Risiko an perioperativen Frakturen, bei ähnlichen Raten an aseptischen Lockerungen und Infektionen [18]. Obwohl die Modularität eine potenzielle Schwachstelle darstellt, konnte in kurz- und mittelfristigen Ergebnissen gezeigt werden, dass es nur in seltenen Fällen (0–2 %) zu Schaftbrüchen kommt [18]. Eine detaillierte Übersicht zu den Ergebnissen ist in Tab. 3 dargestellt.

**Table 3:** 

Autor (Jahr)	*N*	Häufigste Indikation	Defektgröße	Follow-up (Jahre)	Aseptische Lockerung	Schaftmigration > 5 mm	Periprothetische Fraktur	Implantatversagen	Revisionsfreies Überleben
*Nichtmodulare Revisionsschäfte*									
Schnurr et al. 2017 [56]	1090	N/A	N/A	6	0,5 %	N/A	0,4 %	0 %	97,6 %
Herry et al. 2019 [27]	116	Aseptische Lockerung (82 %)	N/A	10	0 %	3 %	2,6 %	0 %	95,7 %
Gabor et al. 2020 [23]	157	Periprothetische Frakturen (22,9 %)	I bis IV	1	0 %	11,87 %	3,8 %	0 %	96,2 %
Saunders et al. 2020 [55]	254	Aseptische Lockerung (75 %)	I bis IIIb	5,2	1,2 %	11 %	11,4 %	0,3 %	94,8 %
*Modulare Revisionsschäfte*									
Wirtz et al. 2014 [66]	163	Aseptische Lockerung (95 %)	I bis III	5,7	0 %	12 %	3 %	0 %	97 %
Schnurr et al. 2017 [56]	314^a^ 230^b^	N/A	N/A	6 6	0,6 % 0,8 %	N/A	0 % 0,4 %	4,4 % 0 %	90,6 % 95,4 %
Abdel et al. 2017 [2]	519	Aseptische Lockerung (100 %)	I bis IV	4,5	1,18 %	2,4 %	13 %	0,2 %	96 %
Fink et al. 2019 [21]	202	N/A	II und IIIa	7,4	N/A	2,1–3,3 %	0 %	0,5 %	N/A

^a^Modularer Schaft mit Titan Hals

^b^Cobalt Chrom Hals

Langzeitdaten zu zementierten Schäften zeigen, dass hier im Vergleich zu zementfreien Schäften eine erhöhte Rate an Re-Revisionen aufgrund einer aseptischen Lockerung besteht [39, 61]. Im kurz- und mittelfristigen Follow-up zeigen zementierte Schäfte allerdings bei älteren Patienten eine niedrige Revisionsrate, welche durch ein geringeres Risiko für eine periprothetische Fraktur zu erklären ist [1, 61]. Der Einsatz von „impaction bone grafting“ in Kombination mit zementierten Schäften zeigt bei ausgeprägten kavitären Femurdefekten gute mittel- bis langfristige Ergebnisse mit Revisionsraten unter 10 % nach 10–15 Jahren. Bei festsitzendem Zementmantel zeigt das Vorgehen einer Zement-in-Zement-Revision ebenfalls gute mittelfristige Ergebnisse, mit einem revisionsfreien Überleben von 94 % nach 6 Jahren [43]. Trotzdem sollte dieses Vorgehen nur in Ausnahmesituationen bei Patienten mit geringem Anspruch durchgeführt werden. Größere Studien mit entsprechend langen Standzeiten fehlen derzeit noch [42]. Tab. 4 fasst die Ergebnisse zu zementierten Revisionsschäften zusammen.

**Table 4:** 

Autor (Jahr)	*N*	Häufigste Indikation	Defektgröße	Follow-up (Jahre)	Aseptische Lockerung	Schaftmigration > 5 mm	Periprothetische Fraktur	Infektion	Revisionsfreies Überleben
*Zementiert*									
Lie et al. 2005 [39]	2011	N/A	N/A	3,4–5	N/A	N/A	N/A	N/A	91,3 %
Tyson et al. 2019 [61]	1328	Aseptische Lockerungen (100 %)	N/A	4,5	4,9 %	N/A	1 %	1,4 %	88 %
*Zementiert mit „impaction bone grafting“*									
Ornstein et al. 2009 [49]	1305	Aseptische Lockerungen (88,7 %)	N/A	5–18	1 %	N/A	1,3 %	1,2 %	94 %
Wilson et al. 2016 [65]	705	Aseptische Lockerungen (62 %)	N/A	10	1 %	15 %	3,3 %	3,4 %	89,2 %
*Zement-in-Zement*									
Stefanovich et al. 2014 [57]	44	Pfannen Lockerung (63 %)	N/A	5,3	0 %	N/A	0 %	2,3 %	95,2 %
Cnudde et al. 2017[14]	1179	Aseptische Lockerung (78 %)	N/A	6,7	2,2–2,8 %	N/A	0,6–1,1 %	1,2–2,8 %	90,6–91,4 %
Woodbridge et al. 2019 [68]	166	Pfannen Lockerungen (65 %)	N/A	8,1	1,8 %	N/A	1,8 %	3,6 %	88,9 %

Die häufigsten Komplikationen von Megaprothesen (proximaler und totaler Femurersatz) sind die Luxation, die PPI, und die aseptische Lockerung, wobei einzelne Studien oft nur kleine Kohorten aufweisen. Ein systematisches Review von Korim et al. konnte 14 Studien einschließen, in denen insgesamt 356 Patienten untersucht wurden. Hier waren die Luxation (16 %), gefolgt von der PPI (8 %) und der aseptischen Lockerung (3 %) die führenden Revisionsgründe. Weiterhin traten PPF (1 %) und Implantatbrüche (0,5 %) auf. Bei einem mittleren Follow-up von 2–5,7 Jahren betrug das revisionsfreie Überleben 83 % [34]. Ein Review von DeRogatis et al. mit insgesamt 6 Studien und insgesamt 277 Patienten zeigte Infektionsraten von 4–44 %, Luxationsraten von 6–38 % und mechanisches Versagen bei 3–11 % der Patienten [17]. Eine Übersichtsarbeit von Putman et al. zeigt eine ähnliche Rate an Komplikationen und ein revisionsfreies Überleben nach 10 Jahren von 70–86 % [51]. Zusammenfassend zeigen Megaprothesen deutlich höhere Komplikationsraten als Revisionsschäfte und sollten aus unserer Sicht nur als „salvage-procedure“ genutzt werden. (Tab. 5).

**Table 5:** 

Autor (Jahr)	*N*	Häufigste Indikation	Defektgröße	Follow-up (Jahre)	Aseptische Lockerung	Luxation	Infektion	Periprothetische Fraktur	Revisionsfreies Überleben
*Proximaler Femurersatz*									
Al-Taki et al. 2011 [5]	36	Aseptische Lockerung (42 %)	IIIB	3,2	5 %	8 %	3 %	0 %	90,5 %
Viste et al. 2017 [63]	44	Aseptische Lockerung (34 %)	IIIB bis IV	6	2,2 %	13,6 %	4,5 %	4,5 %	95,5 %
De Martino et al. 2019 [16]	41	Periprothetische Infektion (42 %)	IIIB bis IV	5	5 %	5 %	7 %	5 %	78 %
*Totaler Femurersatz*									
Friesecke et al. 2005 [22]	100	Periprothetische Frakturen (39 %)	N/A	5	3 %	6 %	13 %	0 %	79 %
Lombardi et al. 2006 [41]	75	N/A	N/A	3,5	3 %	9 %	15 %	1,3 %	69,4 %
Putman et al. 2018 [51]	29	Aseptische Lockerungen (41 %)	N/A	6	0 %	7 %	28,6 %	0 %	79 %

## Fazit für die Praxis

- Die Schaftrevision ist technisch anspruchsvoll und wird häufig unterschätzt.
- Das Vorhandensein von Spezialinstrumentarien, die richtige Zugangswahl, eine schonenden Implantatentfernung sowie ausreichend Erfahrung mit dem Revisionssystem sind elementar für den Therapieerfolg.
- Die Nutzung eines therapeutischen Algorithmus ist hilfreich, um reproduzierbare Ergebnisse zu erzielen und die Komplikationsrate auf ein Minimum zu reduzieren.
- Die häufig komplexen Fälle sollten an spezialisierten Schwerpunktzentren durch erfahrene Revisionsendoprothetiker behandelt werden.

## Abkürzungen

ARMD: „adverse reactions to metal debris“
MARS-MRT: MRT mit Metall-Artefakt-Suppression
PPF: periprothetische Fraktur
PPI: periprothetische Infektion
